# An ancient selective sweep linked to reproductive life history evolution in sockeye salmon

**DOI:** 10.1038/s41598-017-01890-2

**Published:** 2017-05-11

**Authors:** Andrew J. Veale, Michael A. Russello

**Affiliations:** 1 Department of Biology, The University of British Columbia, Okanagan Campus, 3247 University Way, Kelowna, British Columbia V1V 1V7 Canada; 2 Department of Zoology, University of Otago, 340 Great King Street, Dunedin, 9016 New Zealand

## Abstract

Study of parallel (or convergent) phenotypic evolution can provide important insights into processes driving sympatric, ecologically-mediated divergence and speciation, as ecotype pairs may provide a biological replicate of the underlying signals and mechanisms. Here, we provide evidence for a selective sweep creating an island of divergence associated with reproductive behavior in sockeye salmon (*Oncorhynchus nerka*), identifying a series of linked single nucleotide polymorphisms across a ~22,733 basepair region spanning the leucine-rich repeat-containing protein 9 gene exhibiting signatures of divergent selection associated with stream- and shore-spawning in both anadromous and resident forms across their pan-Pacific distribution. This divergence likely occurred ~3.8 Mya (95% HPD = 2.1–6.03 Mya), after sockeye separated from pink (*O. gorbuscha*) and chum (*O. keta*) salmon, but prior to the Pleistocene glaciations. Our results suggest recurrent evolution of reproductive ecotypes across the native range of *O. nerka* is at least partially associated with divergent selection of pre-existing genetic variation within or linked to this region. As sockeye salmon are unique among Pacific salmonids in their flexibility to spawn in lake-shore benthic environments, this region provides great promise for continued investigation of the genomic basis of *O. nerka* life history evolution, and, more broadly, for increasing our understanding of the heritable basis of adaptation of complex traits in novel environments.

## Introduction

Understanding how and why populations diverge into new species or ecotypes are principal goals of evolutionary biology^1^. Of particular interest are the processes driving sympatric, ecologically-mediated divergence and speciation^2^. Recent studies have begun exploring the genetic bases of these processes, detecting genome-wide changes associated with divergent local adaptation^3, 4^, and the specific genes that underlie reproductive isolation^5, 6^.

Parallel (or convergent) phenotypic evolution, the repeated independent emergence of a specific phenotype associated with a particular habitat, can provide important insights into ecological divergence, as each ecotype pair provides a replicate of the underlying signals and mechanisms^4^. When similar environmental pressures and associated selective pressures occur in different populations within a species range, parallel evolution may lead to similar phenotypic changes^7, 8^ that may have the same underlying genetic basis^9, 10^. While such parallel divergent adaptation may originate from novel mutations in the same gene(s), it is often attributed to changes in frequencies of existing alleles^11–13^. A classic example of selection occurring on pre-existing genetic variation comes from the parallel evolution of freshwater forms of three-spined stickleback (*Gasterosteus aculeatus*), where anciently derived alleles in a gene responsible for armor reduction (ectodysplasin-A) have been recurrently selected in multiple freshwater populations^12^.

Studies of the genomic bases of local adaptation have been facilitated by the advent of high-throughput genotyping methods that allow for the identification and genotyping of thousands of genetic polymorphisms throughout the genome enabling population genomic and association studies in non-model organisms^14–16^. Such data further allow for the investigation of the genetics of adaptation through divergence mapping, where large suites of markers are screened for signatures of divergent selection among ecologically distinct populations^17–20^. The key to this approach is that substitutions physically linked to a beneficial allele will ‘hitchhike’, creating a region of lower diversity of fixed alleles in a process known as a ‘selective sweep’^21, 22^. These linked loci will then show higher differentiation between populations than neutral unlinked loci^23^.

Salmonids are an exemplary taxonomic group to study the genetic bases for ecologically-driven, sympatric divergence because abundant environmental variation among river drainages, combined with precise natal homing, create great potential for differential local adaptation^24^. Sockeye salmon, in particular, exhibit tremendous life history and morphological variation, with the repeated parallel evolution of several morphologically and ecologically divergent ecotypes linked to migratory and spawning behaviour^24–26^. All sockeye salmon spawn and spend their early life in freshwater, with anadromous ecotypes then migrating out to sea, and resident ecotypes (kokanee) remaining in freshwater lakes throughout their lifecycle^26, 27^. While the kokanee phenotype is similar across catchments, kokanee populations are polyphyletic, having evolved multiple times from anadromous sockeye salmon through independent postglacial freshwater colonization events^25, 26, 28, 29^.

Both anadromous sockeye salmon and kokanee can be further subdivided into reproductive ecotypes, with each population exhibiting a specific spawning habitat preference. These include the classical ‘stream (or river)-spawning’ ecotypes, ‘shore (or beach)-spawning’ ecotypes that spawn on the shallow submerged shorelines of lakes or island beaches, and ‘black’ kokanee that also spawn on the lake benthos, but at depths down to 70 m below the lake surface^30^. This variability in spawning habitat preference is unique to sockeye among Pacific salmon, with all other species in the genus spawning in streams^31^. In some lakes, multiple reproductive ecotypes co-occur, while in others only one may be present. Divergence between shore- and stream-spawning ecotypes can occur rapidly, with reintroduced sockeye salmon observed to form distinct, reproductively isolated populations of shore- and stream-spawning ecotypes in less than 13 generations^32, 33^. While it is possible that such divergence occurs due to philopatry or learned spawning habitat preferences, we predict there may be underlying genetic mechanisms behind ecotype divergence, potentially involving ‘speciation genes’^34^.

We recently conducted a landscape genomics study employing restriction-site associated DNA sequencing (RADseq) of paired population samplings of migratory (resident versus anadromous) and reproductive (shore- versus stream-spawning kokanee) ecotypes sampled from seven lakes and two rivers spanning three catchments (Columbia, Fraser, and Skeena drainages) in British Columbia, Canada^35^. We identified 334 outlier loci associated with life history variation, one of which was shared in both magnitude and direction of differentiation across all sampled lakes containing sympatric shore- and stream-spawning kokanee (R68810)^35^. The 100 base pair (bp) RAD tag containing this SNP mapped to leucine-rich repeat-containing protein 9 (LRRC9) in both the rainbow trout and Atlantic salmon genomes (*Oncorhynchus mykiss* chromosome 29, *Salmo salar* chromosome 9). LRRC proteins are involved in gene expression and participate in many biologically important processes, such as enzyme inhibition, hormone–receptor interactions, cell adhesion and cellular trafficking^36^. Furthermore, they have been associated with immunotoxicity in different fish species^37, 38^. Interestingly, this SNP was also recorded as highly divergent in a recent study by Nichols *et al*.^39^ between shore-spawning sockeye salmon and stream-spawning kokanee in Redfish Lake, Idaho, USA within the Snake River catchment, a tributary of the Columbia River. No other SNP was identified as being as significantly divergent between any pair of shore- or stream-spawning ecotypes in either of these studies, or in a recently published study comparing beach- and river-spawning sockeye salmon from Alaska (which did not include this locus)^40^. Furthermore, no other SNP has been recorded across multiple comparisons of shore- and stream-spawning ecotypes across these studies^39, 40^.

To test whether variation at this locus underlies sockeye salmon reproductive life history variation across the pan-Pacific distribution of the species, here we genotyped 1519 anadromous sockeye salmon and resident kokanee from 47 shore- and stream-spawning populations from Russia, Alaska and Canada. In addition, we sequenced approximately 23,000 bp spanning the entire LRRC9 gene to further characterize the surrounding genomic region and validate the role and history of divergent selection underlying reproductive ecotype divergence within sockeye salmon.

## Results

### Range-wide ecotype genotyping

**Figure 1:**
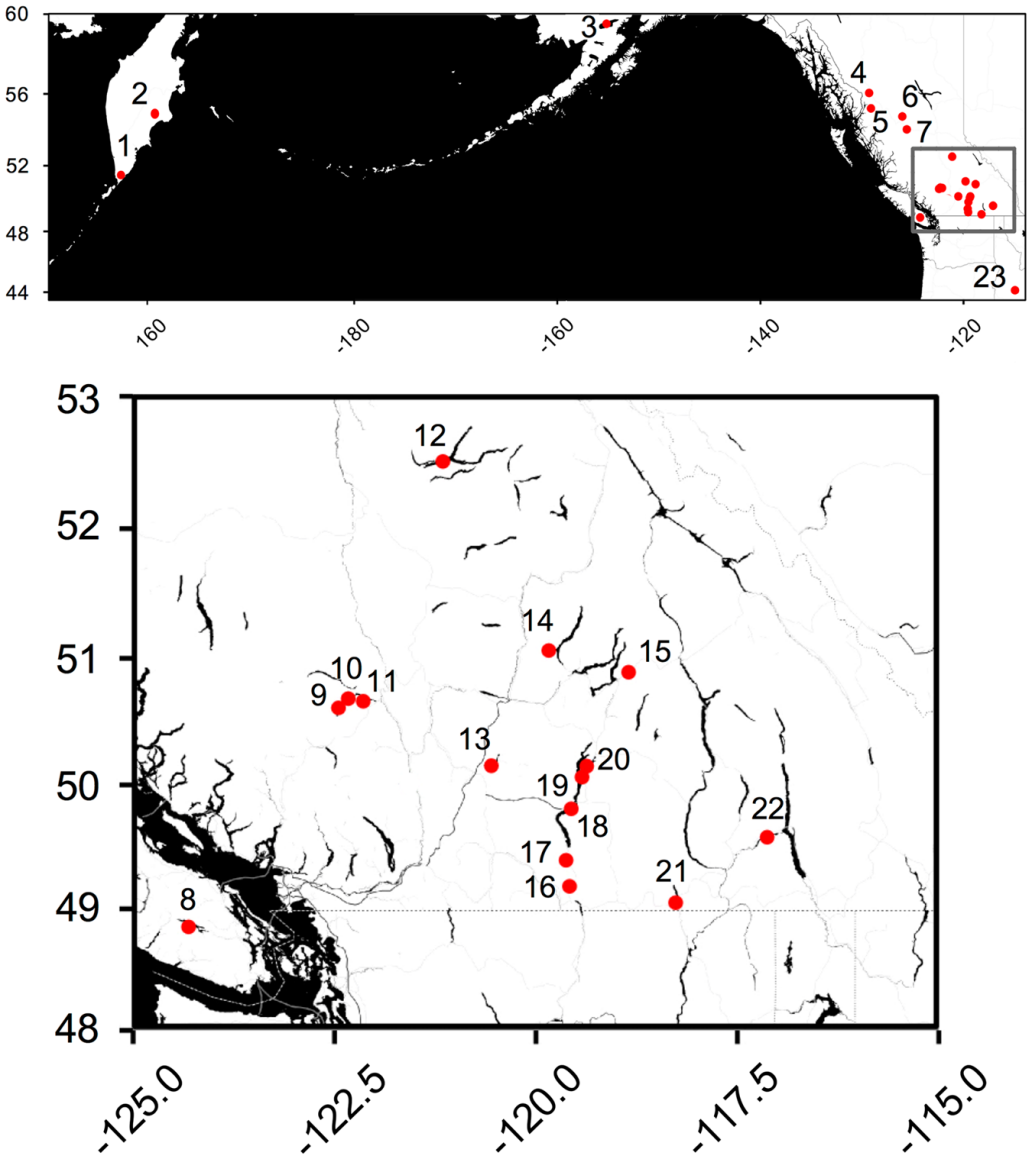
Map of sampling localities included in this study across the range of *Oncorhynchus nerka*. Locality numbers as in Table 1. The map was generated in R^67^ using the ggmap package^68^ with map tiles by Stamen Design (www.stamen.com), under CC BY 3.0 (creativecommons.org/licenses/by/3.0/) and data by OpenStreetMap (www.openstreetmap.org/), under ODbL (www.openstreetmap.org/copyright).

**Figure 2:**
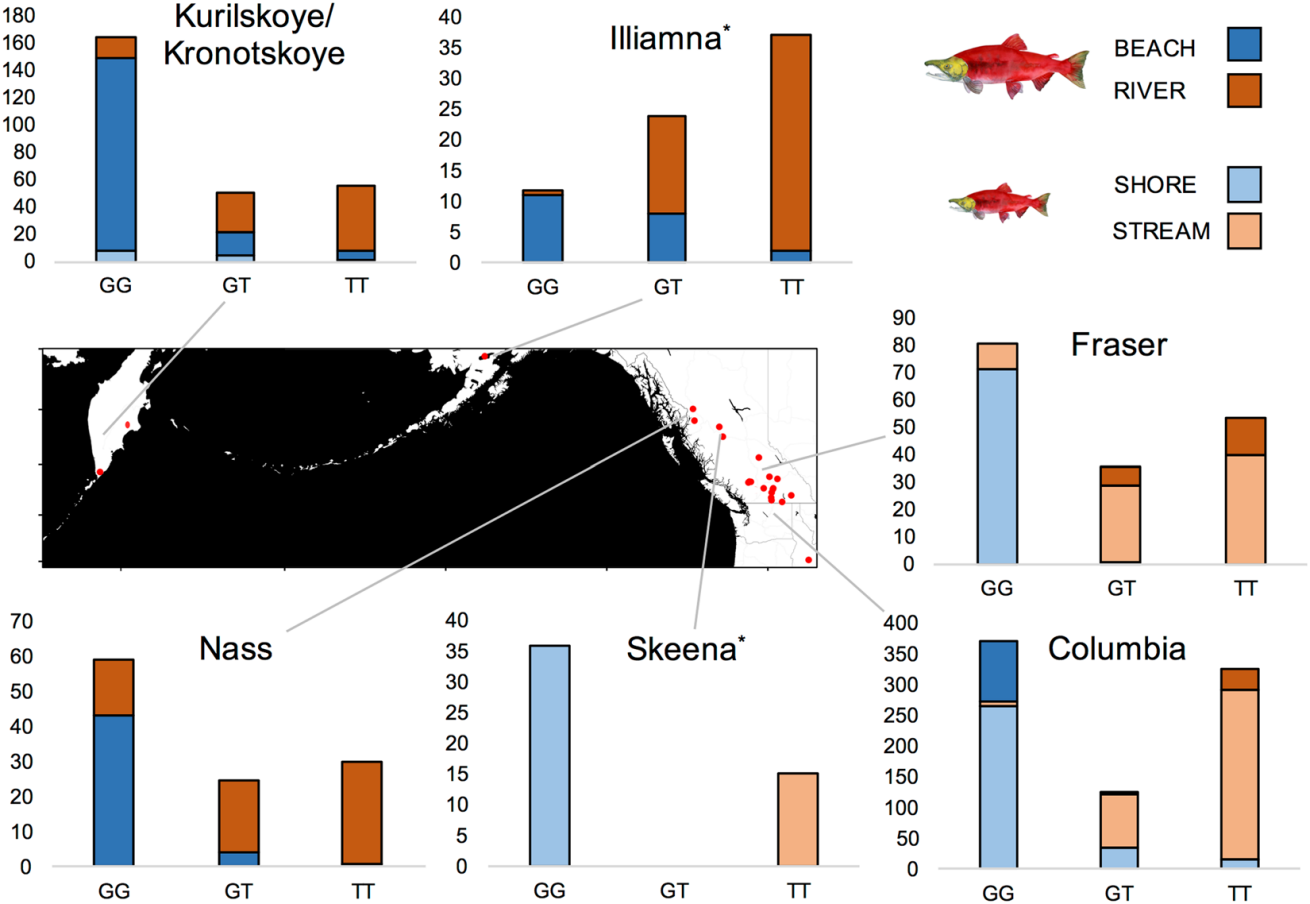
Genotype frequencies at One_LRRC9_68810 of *Oncorhynchus nerka* migratory and reproductive ecotypes across the major catchments sampled in this study. Plot shading indicates the proportion of each genotype per ecotype per catchment, with colors indicated in the inset legend and general location shown on the map. The Illiamna plot includes all localities within the catchment indicated in Fig. 1 and Table 1 except for the island beach-spawning sockeye salmon samples (Fuel Dump Island and Woody Island). The Skeena plot includes all localities within the catchment indicated in Fig. 1 and Table 1 except for the Drew Creek samples from Tchesinkut Lake (see Discussion in main text for more details). Genotype frequencies from the Vancouver Island catchment are also not shown, as we only have three samples from a single site (Cowichan Lake). All three of these shore-spawning kokanee possessed the predicted GG genotype. Genotype frequencies for all sample sites are given in Table S3. The map was generated in R^67^ using the ggmap package^68^ with map tiles by Stamen Design (www.stamen.com), under CC BY 3.0 (creativecommons.org/licenses/by/3.0/) and data by OpenStreetMap (www.openstreetmap.org/), under ODbL (www.openstreetmap.org/copyright).

Genotypic data collected using a newly developed TaqMan™ assay (One_LRRC9_68810; Table S1) targeting this SNP showed directional divergence in both anadromous sockeye salmon and resident kokanee across the natural range of *O. nerka* in Russia, Alaska and Canada (Table 1 and Figs 1 and 2). In general, the ‘G’ allele was most prevalent in shore-spawning sockeye salmon and kokanee populations, while the ‘T’ allele dominated in stream-spawning populations (Table 1 and Fig. 2). Notable exceptions were two shore-spawning sockeye salmon sites along island beaches in Illiamna Lake, Alaska and one shore-spawning kokanee site in the West Arm of Kootenay Lake, BC, all of which exhibited a higher frequency of the ‘T’ allele than observed in other shore-spawning populations. In addition, kokanee sampled while spawning near the mouth of Drew Creek in Tchesinkut Lake were fixed for the ‘G’ allele, identical to all sampled shore-spawning individuals from this system.

**Table 1:** Range-wide sampling information of *Oncorhynchus nerka*.

Map	Waterbody	Population	Region	Catchment	Ecotype		Sample	Allele (G)	References
#					Migratory	Reproductive	Size	Frequency	
1	Kurilskoye Lake	Close North	Kamchatka, Russia	Kurilskoye	Sockeye	Shore	24	0.90	Beacham *et al*.^69^
		Far North	Kamchatka, Russia	Kurilskoye	Sockeye	Shore	23	0.87	Beacham *et al*.^69^
		Gavrushka Bay	Kamchatka, Russia	Kurilskoye	Sockeye	Shore	24	0.71	Beacham *et al*.^69^
		Khakitzin Bay	Kamchatka, Russia	Kurilskoye	Sockeye	Shore	24	0.94	Beacham *et al*.^69^
		Oladochnaya Bay	Kamchatka, Russia	Kurilskoye	Sockeye	Shore	24	0.96	Beacham *et al*.^69^
		Ozernaya	Kamchatka, Russia	Kurilskoye	Sockeye	Shore	22	1.00	Beacham *et al*.^69^
		South Bay	Kamchatka, Russia	Kurilskoye	Sockeye	Shore	24	1.00	Beacham *et al*.^69^
		Etamink River	Kamchatka, Russia	Kurilskoye	Sockeye	Stream	24	0.56	Beacham *et al*.^69^
		Gavrushka River	Kamchatka, Russia	Kurilskoye	Sockeye	Stream	24	0.27	Beacham *et al*.^69^
		Kirushutk River	Kamchatka, Russia	Kurilskoye	Sockeye	Stream	24	0.27	Beacham *et al*.^69^
		Vichenkiya River	Kamchatka, Russia	Kurilskoye	Sockeye	Stream	21	0.17	Beacham *et al*.^69^
2	Kronotsky Lake	Kronotsky Lake	Kamchatka, Russia	Kronotskoye	Kokanee	Shore	12	0.75	Taylor *et al*.^29^
3	Illiamna Lake	Fuel Dump Island	Alaska, USA	Illiamna	Sockeye	Shore	14	0.32	Beacham *et al*.^69^
		Knutson Bay	Alaska, USA	Illiamna	Sockeye	Shore	21	0.71	Beacham *et al*.^69^
		Woody Island	Alaska, USA	Illiamna	Sockeye	Shore	20	0.28	Beacham *et al*.^69^
		Chinkelyes Creek	Alaska, USA	Illiamna	Sockeye	Stream	11	0.14	Beacham *et al*.^69^
		Gibraltar Creek	Alaska, USA	Illiamna	Sockeye	Stream	21	0.24	Beacham *et al*.^69^
		Copper River	Alaska, USA	Illiamna	Sockeye	Stream	20	0.13	Beacham *et al*.^69^
4	Mezadin Lake	Meziadin Beach	British Columbia, Canada	Nass	Sockeye	Shore	48	0.94	Beacham *et al*.^69^
		Tintina Creek	British Columbia, Canada	Nass	Sockeye	Stream	19	0.50	Beacham *et al*.^69^
		Hanna Creek	British Columbia, Canada	Nass	Sockeye	Stream	24	0.54	Beacham *et al*.^69^
5	Gingit River	Gingit Creek	British Columbia, Canada	Nass	Sockeye	Stream	23	0.17	Beacham *et al*.^69^
6	Babine Lake	Pierre Creek	British Columbia, Canada	Skeena	Kokanee	Stream	15	0.00	Taylor *et al*.^29^
7	Tchesinkut Lake	Tchesinkut Lake	British Columbia, Canada	Skeena	Kokanee	Shore	36	1.00	Frazer & Russello^42^
		Drew Creek	British Columbia, Canada	Skeena	Kokanee	Stream	36	1.00	Frazer & Russello^42^
8	Cowichan Lake	Cowichan Lake	British Columbia, Canada	Vancouver Island	Kokanee	Shore	3	1.00	Taylor *et al*.^29^
9	Anderson-Seton Lakes	Anderson Lake	British Columbia, Canada	Fraser	Kokanee	Shore	22	0.98	Moreira & Taylor^30^
10		Portage Creek	British Columbia, Canada	Fraser	Sockeye	Stream	20	0.18	Moreira & Taylor^30^
11		Seton Lake	British Columbia, Canada	Fraser	Kokanee	Shore	23	1.00	Moreira & Taylor^30^
12	Quesnel Lake	Quesnel Lake	British Columbia, Canada	Fraser	Kokanee	Shore	27	1.00	Taylor *et al*.^29^
13	Nicola Lake	Upper Nicola River	British Columbia, Canada	Fraser	Kokanee	Stream	24	0.19	Frazer^70^
14	Adams Lake	Momich Creek	British Columbia, Canada	Fraser	Kokanee	Stream	24	0.23	Taylor *et al*.^29^
		Sinmax Creek	British Columbia, Canada	Fraser	Kokanee	Stream	21	0.55	Taylor *et al*.^29^
15	Shuswap Lake	Eagle River	British Columbia, Canada	Fraser	Kokanee	Stream	9	0.28	Taylor *et al*.^29^
16	Skaha Lake	Okanagan River	British Columbia, Canada	Columbia	Sockeye	Stream	33	0.02	Veale & Russello^71^
17		Penticton Channel	British Columbia, Canada	Columbia	Kokanee	Stream	19	0.08	Veale & Russello^71^
18	Okanagan Lake	Okanagan Lake	British Columbia, Canada	Columbia	Kokanee	Shore	144	0.99	Lemay & Russello^59^
		Mission Creek	British Columbia, Canada	Columbia	Kokanee	Stream	136	0.10	Lemay & Russello^59^
19	Wood Lake	Wood Lake	British Columbia, Canada	Columbia	Kokanee	Shore	48	0.92	Frazer & Russello^42^
		Middle Vernon Creek	British Columbia, Canada	Columbia	Kokanee	Stream	48	0.28	Frazer & Russello^42^
20	Kalmalka Lake	Kalmalka Lake	British Columbia, Canada	Columbia	Kokanee	Shore	32	0.95	Veale & Russello^71^
		Coldstream Creek	British Columbia, Canada	Columbia	Kokanee	Stream	32	0.23	Veale & Russello^71^
21	Christina Lake	Christina Lake	British Columbia, Canada	Columbia	Kokanee	Shore	48	1.00	Frazer^70^
		Sanders Creek	British Columbia, Canada	Columbia	Kokanee	Stream	48	0.07	Frazer^70^
22	Kootenay Lake	Kootenay Lake (West Arm)	British Columbia, Canada	Columbia	Kokanee	Shore	46	0.37	Lemay & Russello^72^
		Duhamel Creek (West Arm)	British Columbia, Canada	Columbia	Kokanee	Stream	32	0.19	Lemay & Russello^72^
		Meadow Creek (North Arm)	British Columbia, Canada	Columbia	Kokanee	Stream	22	0.11	Morbey *et al*.^73^
23	Redfish Lake^*^	Redfish Lake	Idaho, USA	Columbia	Sockeye	Shore	99	1.00	Nichols *et al*.^39^
		Fishhook Creek	Idaho, USA	Columbia	Kokanee	Stream	34	0.10	Nichols *et al*.^39^

^*^Allele frequency data from Nichols *et al*.^39^.

Five within-lake reproductive ecotype pairs spanning multiple catchments had >99% correct assignment to reproductive ecotype under a straight Mendelian assignment rule (GG = shore-spawning, GT or TT = stream-spawning), including Okanagan Lake kokanee shore- and stream-spawners, Christina Lake kokanee shore- and stream-spawners, Anderson Lake black kokanee/Portage Creek sockeye salmon, Seton Lake black kokanee/Portage Creek sockeye salmon, and Redfish Lake shore-spawning sockeye salmon/Fishhook Creek stream-spawning kokanee. In these systems, GG was recorded only once across the 238 stream-spawning individuals genotyped, and GT was only recorded four times within the 336 shore-spawning individuals (TT was never observed among shore-spawners) (Fig. 2 and Table S2). For the other two clearly differentiated co-occurring kokanee ecotype-pairs in Wood and Kalamalka Lakes, assignment accuracy using this rule was also high at >90%.

### Flanking region sequencing

Sanger sequencing of 744 bp flanking this SNP in Okanagan Lake kokanee (Columbia River drainage), Anderson Lake kokanee and Portage Creek sockeye salmon (Fraser River drainage), revealed three additional SNPs in full linkage, suggesting this region was ancestrally inherited for both alleles in these populations spanning different river catchments.

**Figure 3:**
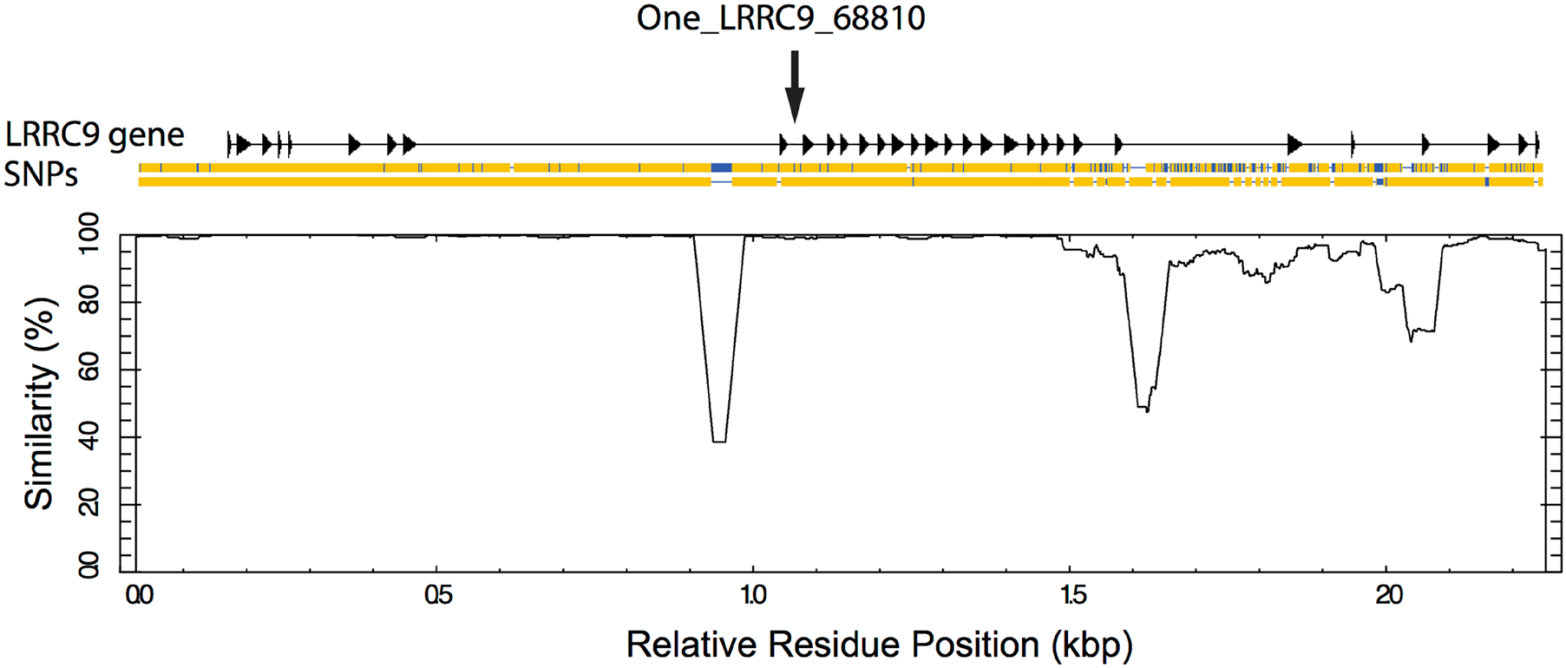
Sequence similarity between the shore- and stream-spawning sequences for the LRRC9 genomic region of *Oncorhynchus nerka* from Okanagan Lake kokanee. Exons for the LRRC9 gene shown at top as arrows, introns are shown as the intervening line, along with the One_LRRC9_68810 SNP location. SNPs are shown as blue vertical lines and indels shown as gaps in the sequence, with identical sequence in orange. “Shore-allele” sequence: top, “stream-allele” sequence: bottom. Similarity is defined as the percentage of identical sites within a 500 bp window centred on this position.

To more broadly characterize this region, we successfully sequenced 22,773 bp flanking the One_LRRC9_68810 SNP from eight individuals each carrying homozygous genotypes of Okanagan Lake shore- and stream-spawning kokanee, respectively, including the entire LRRC9 gene (Genbank accession KY681681-KY681682). These two sequences were 4.6% divergent from each other, with 181 fixed differences including 23 multiple bp indels up to 308 bp long between the ‘shore-spawning’ and ‘stream-spawning’ alleles, suggesting the genomic region around LRRC9 has undergone a significant selective sweep (Fig. 3). Notably, the level of divergence between alleles increased markedly downstream (towards the 3′ end of the LRRC9 gene) (Fig. 3). Variation within each ecotype was far lower, with ten variable sites within the shore-spawning population, and nine variable sites within the stream-spawning population. While five SNPs were identified within the coding regions of the LRRC9 gene, we found no non-synonymous mutations between the ‘shore-spawning’ and ‘stream-spawning’ alleles.

### Divergence timing of the two alleles

**Figure 4:**
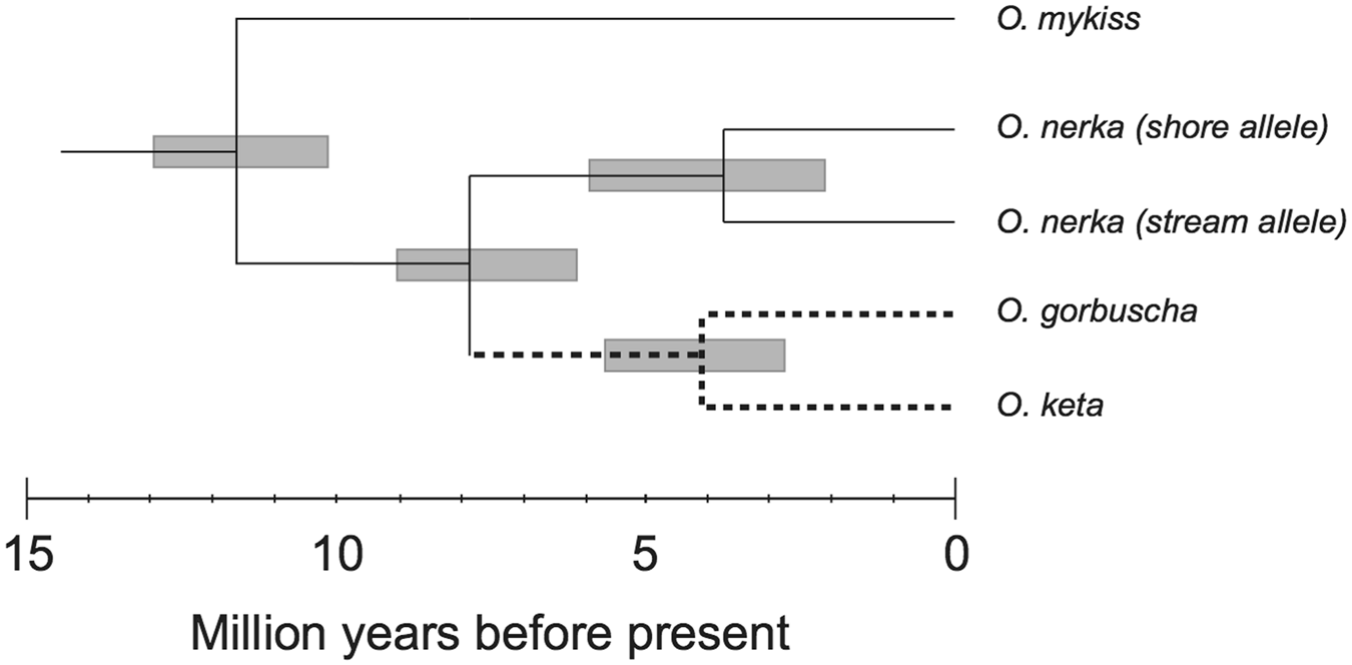
Time to most recent common ancestor for the shore- and stream-spawning alleles in LRRC9 as derived from BEAST^64^. All other divergence timings are based on a previously published molecular clock^41^.

Using an estimated rainbow trout/sockeye salmon divergence time of approximately 11.4 million years ago (Mya)^41^, BEAST analysis revealed ‘shore-spawning’ and ‘stream-spawning’ alleles diverged from each other approximately 3.8 Mya (95%HPD = 2.1–6.03 Mya) (Fig. 4).

## Discussion

Our results show the recurrent evolution of reproductive ecotypes across the native range of *O. nerka* is at least partially associated with divergent selection of pre-existing genetic variation within or linked to the region surrounding the LRRC9 gene. Although no non-synonymous changes were detected between LRRC9 ‘shore-spawning’ and ‘stream-spawning’ alleles, this does not preclude changes in splice sites or regulatory regions of the gene. Differentiation at this locus between shore- and stream-spawning populations was extremely high even when there was minimal neutral population differentiation. For example, pairwise *F*_*ST*_ for One_LRRC9_68810 was 0.92 among reproductive ecotypes of Okanagan Lake kokanee despite the population genomic *F*_*ST*_ of 0.008 based on 6,234 neutral SNPs^35^.

The few failures of this SNP to discriminate between ecotypes are likely explained by a combination of uncertainties in sampling, incomplete ecotype differentiation, and/or recombination events separating the SNP from the related genomic changes. For instance, in Tchesinkut Lake, the vast majority of individuals spawn on the shores of an island where the “shore-spawners” were originally sampled in 2010^42^. The “stream-spawners” were sampled at an outflow and Drew Creek, however, the latter spawning activity only occurred once Ministry personnel cleared the mouth allowing kokanee access^42^. These observations, in tandem with the complete lack of divergence at 6,234 neutral SNPs, strongly suggest there may not be distinct ecotypes in Tchesinkut lake^35^. The other exceptions where stream-spawning populations had elevated levels of the G allele (Sinmax Creek kokanee, Tintina and Hanna Creek sockeye salmon) are immediately adjacent to shore-spawning sites in gravel near the creek mouths^43^. Finally, we obtained samples from three sockeye salmon beach-spawning sites in Illiamna Lake in Alaska, two on island beaches (Fuel Dump Island; Woody Island) and one mainland beach (Knutson Bay). While genotype frequencies within the mainland beach-spawning site were consistent with range-wide patterns (Fig. 2 and Table S2), the island-beach spawning sites showed relatively uniform genotype frequencies (Table S2). Specific beaches on these islands lack the upwelling groundwater typical of most beach-spawning salmonids^44^, and consequently, may not contain the site-specific olfactory signals to guide returning adults that would be necessary for promoting ecotype differentiation^45^.

In at least five lakes spanning the Columbia and Fraser River drainages (Anderson, Seton, Okanagan, Christina, Redfish), assignment accuracy to reproductive ecotype was >99%. Given our results, it appears that this SNP could be associated with a Mendelian trait, potentially creating a switch to an alternative spawning behaviour. Understanding how such a genetic switch might work in terms of influencing behaviour awaits future breeding and physiological experiments. If this SNP is linked to a gene that causes a change in spawning habitat preference, our data are consistent with the hypothesis that the T (stream) allele is associated with a preference or ability for stream-spawning, while the G (shore) allele could be a loss of function, leading to a lack of spawning habitat preference, enabling spawning anywhere with suitable substrate.

This hypothesis fits well with several other lines of evidence from shore- and stream-spawning populations of *O. nerka*. For example, in Lake Washington, Washington, USA, a reintroduced population of anadromous sockeye salmon diverged into reproductively isolated shore- and stream-spawning populations within 13 generations^32, 33^. In this system, both populations were derived from the same hatchery stock, and the population solely exhibited stream-spawning behaviour initially; shore-spawning was first recorded over 17 years after their introduction. The hatchery stock was derived from both stream-spawning and shore-spawning individuals^32^. This rapid heritable divergence into two spawning ecotypes matches the predictions for a previously existing polymorphism in a gene or genes that influence spawning behaviour, with shore-spawning recessive to stream-spawning. Similarly, in a study of anadromous sockeye salmon in Little Togiak Lake, Alaska where individuals genetically identified as coming from one population spawned in the alternate habitat, straying was rare and asymmetrical, primarily with individuals from the stream-spawning population using shore habitats^46^. These findings are consistent with the predicted pattern of a recessive allele promoting shore-spawning behaviour.

Sockeye salmon are known to have survived the late Wisconsin glaciation in several refugia, including areas south of the ice sheets such as the Columbia River, arid northern areas that remained largely free of ice in Beringia (region connecting Kamchatka and much of western Alaska), and in small inland mountain refugia where glaciers impeded access to the sea^29^. Sockeye salmon and kokanee descended from all of these refugia exhibit variability at this locus and evidence for divergent selection between ecotypes; therefore divergence between the two alleles must significantly predate the last glacial maximum. We estimate that the ‘shore-spawning’ and ‘stream-spawning’ alleles most likely diverged from each other around 3.8 Mya in the Pliocene, after sockeye separated from pink (*O. gorbuscha*) and chum (*O. keta*) salmon, but prior to the Pleistocene glaciations. While there are many simplifying assumptions for estimating the time to the most recent common ancestor for this region, particularly as it has likely been under selection, our divergence time estimate does highlight the great age of these alleles, both of which have been maintained in populations across the range of the species.

This example of recurrent selection of pre-existing variants in the population as a source for ecologically-driven sympatric divergence closely resembles the parallel evolution of freshwater forms of stickleback^12^. In this system, anadromous populations carry the anciently derived ‘freshwater’ armor genes at low frequency that then repeatedly went to fixation when stickleback colonized similar freshwater environments. Here, while the ‘G’ (shore) allele was uniformly prevalent in shore-spawning populations of both kokanee and sockeye salmon, it was never absent from stream-spawning populations. This suggests that (most) stream-spawning populations carry the ‘G’ allele, which is then strongly selected for as shore-spawning populations form.

### Identifying the gene(s) under divergent selection

The size of the genomic region associated with a selective sweep of a locus under selection is determined by the strength of selection, local rate of recombination, and time since the beneficial mutation arose^21, 47, 48^. Because of these factors, genomic scans for signatures of selection may highlight regions spanning several megabase pairs (Mbp); therefore determining the gene(s) under selection in such cases remains challenging^49^. In a recent review of selective sweeps in cattle breeds, the size of genomic regions showing signals of selection ranged from 8.2 to 948 kilobase pairs (kbp), with a median of 78.7 kbp^50^. In dog breeds, which have a recent history of very strong selective pressures and inbreeding, selective sweeps may be up to 10 Mbp^51^. The most phylogenetically similar species to sockeye salmon with dense SNP panels is the Atlantic salmon (*Salmo salar*). In a recent study of this species, SNPs significantly associated with age at maturity were located in a selective sweep region covering ~370 kbp^52^.

There are 17 other genes within 250 kbp on either side of the LRRC9 gene in the *S. salar* genome (Table S4), and it remains possible that the genetic variation under divergent selection is outside this area, or that there are *O. nerka* structural differences compared to Atlantic salmon where other genes could be closer. While we highlight an ancient selective sweep between alleles closely linked to, and potentially underlying spawning behaviour in this genomic region, we have not yet identified the specific gene(s) linked to this divergent selection. Interestingly, this same genomic region of chromosome 9 in Atlantic salmon has been identified as an island of divergence between *S. salar* genetic clusters that differed in the length of sea migration and age at maturity^53, 54^. Of particular note, Barson *et al*.^53^ found an island of divergence spanning 250 kb centered in this region that was strongly associated with age at maturity. They hypothesized that variation in SIX6, a transcriptional regulator gene and distal forebrain enhancer^55^ that also regulates eye development across multiple taxa^56^, age at maturity in humans^57^, neuro-endocrine and gonad development^58^, might be the cause of this divergence. This gene is 142 kb away from the LRRC9 in Atlantic salmon. Whatever the underlying genetic mechanism, it is noteworthy that this region is associated with local adaptation and population divergence in multiple salmonids. A denser SNP map for sockeye salmon, and/or direct sequencing for several 100 kbp across multiple populations and ecotypes will be required to ascertain the underlying divergently selected gene(s). As it appears that the level of divergence between the ‘shore-spawning’ and ‘stream-spawning’ alleles increases towards the 3′ end (and the SIX6 gene in *S. salar*), this may be the direction to initially explore for the specific target(s) of divergent selection. As sockeye salmon are unique among the Pacific salmonids in their flexibility to spawn in lake-shore benthic environments^31^, this region provides great promise for future investigations of the genomic basis of *O. nerka* life history evolution, and more broadly, for increasing our understanding of the heritable basis of adaptation of complex traits in novel environments. From an applied perspective, this highly informative SNP has immediate utility for informing fisheries management throughout British Columbia^59^ and likely across the entire range.

## Methods

### Sampling

We used previously extracted DNA from 1519 anadromous sockeye salmon and resident kokanee from 47 shore- and stream-spawning populations in Russia, Alaska and Canada (Table 1). All original sampling and experimental procedures were conducted in accordance with institutional, national and international guidelines and regulations as cited within the original published work (Table 1).

### SNP genotyping

We designed a new TaqMan™ assay (One_68810_LRRC9; Table 1) using a previously sequenced 100 bp RAD tag 68810 containing the SNP of interest^35^. SNP genotyping of all samples was performed using this TaqMan™ assay in 6 μl reactions: 2.5 μl TaqMan™ Universal PCR Master Mix (Life Technologies, Carlsbad, CA), 0.25 μl TaqMan™ Genotyping Assay (20x), 1.25 μl H_2_O and 2 μl of 1/10 diluted extracted DNA. Genotyping reactions were performed in 384 well plates using an Applied Biosystems ViiA7™ Real-Time PCR system (Life Technologies, Carlsbad, CA).

### Flanking region sequencing and comparison

We used BLAST-n to locate and align the 100 bp RAD tag 68810 with the Atlantic salmon (*Salmo salar*) ICSASG_V2 (ssa09: 24,748,525–24,748,624) and rainbow trout (*Oncorhynchus myskiss*)^60^ genomes (chrUn_29: 1,729,057–1,729,247). We then aligned ~60 kbp of the flanking genomic regions of these two species centered on the 68810 SNP using a global alignment with open ends, assuming 70% similarity as implemented in Geneious 9.0.5^61^.

Using this alignment (*O. mykiss* chromosome 29, *S. salar* chromosome 9), we designed PCR primers (Table S1) in PRIMER3^62^ to amplify a ~750 bp fragment immediately surrounding the SNP for known homozygotes for four individuals from each of: Anderson Lake black kokanee, Portage Creek stream-spawning sockeye salmon, Okanagan Lake stream-spawning kokanee, and Okanagan Lake shore-spawning kokanee. All PCRs were carried out on an ABI Veriti thermal cycler in 25 μl reactions containing: 20–50 ng of DNA, 10 mM Tris-HCl (pH 8.3), 50 mM KCl, 1.5 mM MgCl_2_, 200 μM dNTPs, 0.5 μM of each primer, 20 μg bovine serum albumin (BSA) and 0.5 U of AmpliTaq Gold DNA polymerase (Applied Biosystems). Cycling conditions were as follows: 95° (5 minutes), 30 cycles of 94° (20 seconds), 57° (30 seconds), 72° (45 seconds), and a final extension of 72 °C (7 minutes). All PCR products were purified by ExoSAP-IT (USB Products, Santa Clara, CA, USA) and Sanger sequenced using an ABI 3130XL Genetic Analyzer (Applied Biosystems).

The resulting sequences of the immediate flanking regions, along with the *S. salar* and *O. mykiss* alignment, were used to design two sets of primers in PRIMER3^62^ for long-range PCRs in each direction targeting two overlapping fragments each of ~11 kbp (total contiguous sequence length of ~21 kbp). Long range PCRs were conducted using the LongAmp® *Taq* PCR kit (NEB) for eight individuals of each homozygous genotype at the 68810 SNP from Okanagan Lake shore- and stream-spawning kokanee, respectively. Each long-range PCR was carried out in 25 μl reactions containing: ~100 ng of template DNA, 60 mM Tris-SO_4_, 20 mM (NH_4_)_2_SO_4_, 2 mM MgSO_4_, 3% Glycerol, 0.06% IGEPAL® CA-630, 0.05% Tween® 20, 300 µM dNTPs, 0.5 μM of each primer, and 5 U LongAmp® *Taq* polymerase. Cycling conditions were as follows: 94° (30 seconds), 30 cycles of 94° (20 seconds), 58° (30 seconds), 65° (12 minutes), and a final extension of 65 °C (10 minutes). These PCR products were purified using a Qiagen MinElute gel extraction kit and individuals for each PCR product were pooled. Sequencing libraries were constructed by shearing the PCR products to ~400 bp and using the Illumina TruSeq DNA kit. Libraries were subsequently sequenced using the Illumina MiSeq PE250 platform. Library construction and sequencing were performed at the McGill University and Génome Québec Innovation Centre, Montréal, Canada.

Obtained sequence reads were assembled using the Geneious 9.0.5^61^ *de novo* assembler, with medium sensitivity, five iterations and a maximum indel size of 1000 bp. The libraries for each ecotype were subsequently combined using a pairwise local alignment and variants detected using a minimum minor allele frequency of 0.25.

As the long-range PCRs did not cover the entire LRRC9, we designed two further primer pairs covering ~1 kbp each (LRRC_FR1 & LRRC_FR2; Table S2) to span the missing portions of the gene to give a final contiguous sequence length of ~23 kbp. All PCRs were carried out using the same individuals in 25 μl reactions containing: 20–50 ng of DNA, 10 mM Tris-HCl (pH 8.3), 50 mM KCl, 1.5 mM MgCl_2_, 200 μM dNTPs, 0.5 μM of each primer, 20 μg BSA and 0.5 U of AmpliTaq Gold DNA polymerase (Applied Biosystems). Cycling conditions were as follows: 95° (5 minutes), 30 cycles of 94° (20 seconds), 56° (30 seconds), 72° (60 seconds), and a final extension of 72 °C (7 minutes). All PCR products were purified by ExoSAP-IT (USB Products, Santa Clara, CA, USA) and Sanger sequenced using an ABI 3130XL Genetic Analyzer (Applied Biosystems).

The concatenated sequences of each sequence (‘shore’ and ‘stream’) were then aligned in Geneious 9.0.5^61^ using a global alignment with free end gaps, assuming a 93% similarity. The percentage differentiation between these aligned sequences was then calculated, and the number of divergent fixed SNPs and indels counted. We used plotcon in EMBOSS^63^ with a window size of 500 bp to display the pattern of differentiation between the sequences. We also translated the resulting LRRC9 DNA sequence using the *S. salar* CDS (LOC106610979) as a guide to identify non-synonymous changes in the coding region. To do this, the five published isoforms of the *S. salar* gene (XM_014210737.1 – XM_014210742.1) were aligned with the two *O. nerka* sequences, and these aligned exons were then translated and aligned with each other to detect any non-synonymous changes between the shore- and stream-alleles – all performed in Geneious 9.0.5^61^.

### Divergence timing

The combined sequences for each ecotype were aligned with each other, and with *O. mykiss* using the Geneious 9.0.5 local alignment (Smith & Waterman) tool assuming 70% similarity. This alignment was used to estimate the time of divergence between the ‘shore-spawning allele’ and the ‘stream-spawning allele’ conducted in BEAST^64^. Analyses were implemented using an HKY substitution model, an estimated divergence time between *O. mykiss* and *O. nerka* of 11.4 Mya (95%CI = 9.8–13 Mya)^41^, a normal distribution prior, and a relaxed lognormal clock with a Yule Birth-Death tree prior. Three independent runs consisting of 100 million generations were conducted, with a 25% burn-in. Outputs were assessed in Tracer^65^ and tree files combined in LogCombiner. Resulting tree files were annotated in TreeAnnotator and visualized in FigTree^66^.

## Electronic supplementary material

Supplementary Information
