# Supplementary material for: An ancient selective sweep linked to reproductive life history evolution in sockeye salmon
Source: Sci Rep. 2017 May 11;7:1747. doi: 10.1038/s41598-017-01890-2 (PMC5431894; doi:10.1038/s41598-017-01890-2)
Supplement: Supplementary file 1 — Supplementary Information [file 41598_2017_1890_MOESM1_ESM.doc]

# An ancient selective sweep linked to reproductive life history evolution in sockeye salmon

# Andrew J Veale^#^ and Michael A Russello^*^

Department of Biology, The University of British Columbia, Okanagan Campus, 3247 University Way, Kelowna, British Columbia, Canada V1V 1V7

**^#^** Current address: Department of Zoology, University of Otago, 340 Great King Street, Dunedin, New Zealand 9016; [andrew.j.veale@gmail.com](mailto:andrew.j.veale@gmail.com)

^*^ Corresponding author: [michael.russello@ubc.ca](mailto:michael.russello@ubc.ca)

**Supplementary Information**

**Table S1.** TaqMan SNP genotyping assay.

| Assay Name | VIC | FAM | VIC Probe Sequence | FAM Probe Sequence | Forward Primer Sequence | Reverse Primer Sequence |
| --- | --- | --- | --- | --- | --- | --- |
| One_LRRC9_68810 | T | G | CTTGGATGAAAAATATCA | CTTGGATGAAACATATCA | TGCAGGGAAATTACTTATGTGATCAT | AAGACAGATCAGTCCGCAGAAAA |

**Table S2**. PCR primers.

| Region | Product Size (bp) | Primer Name | Primer Sequence |
| --- | --- | --- | --- |
| 68810 flanking region | 744 | 68810_F | CCAAGCCACCAAAAGGAACG |
|  |  | 68810_R | ACTCCAGCCAGCGTTTGTAA |
| Left long-range PCR | ~11,000 | LRPCR_Left_F1 | CCGCTTTACCTGTAGATGTAGTTTCC |
|  |  | LRPCR_Left_R1 | GACATAATACTGAGGAGCTGAGGTGA |
| Right long-range PCR | ~11,000 | LRPCR_Right_F1 | CTTTCTGCTGATGGAGCTTGAGAC |
|  |  | LRPCR_Right_R1 | ATAACTCTCACCTGGACCTCTGTGC |
| Far right 1 PCR | 1015 | LRRC9_FR1_F | CACCCATTAATATCACCCGACT |
|  |  | LRRC9_FR1_R | GCAATGACCCACACAGGAGT |
| Far right 2 PCR | 1089 | LRRC9_FR2_F | ATAGGCCGGCAGTGGTACT |
|  |  | LRRC9_FR2_R | CCAGGCAGGTTCATATCCAT |

**Table S3.** Genotype frequencies for range-wide sampling of *Oncorhynchus nerka* at One_LRRC9_68810.

| Map | Waterbody | Population | Catchment | Ecotype |  | Sample |  | |  | |  |
| --- | --- | --- | --- | --- | --- | --- | --- | --- | --- | --- | --- |
| # |  |  |  | Migratory | Reproductive | Size | GG | GT | | TT | |
| 1 | Kurilskoye Lake | Close North | Kurilskoye | Sockeye | Shore | 24 | 19 | 5 | | 0 | |
|  |  | Far North | Kurilskoye | Sockeye | Shore | 23 | 19 | 2 | | 2 | |
|  |  | Gavrushka Bay | Kurilskoye | Sockeye | Shore | 24 | 14 | 6 | | 4 | |
|  |  | Khakitzin Bay | Kurilskoye | Sockeye | Shore | 24 | 21 | 3 | | 0 | |
|  |  | Oladochnaya Bay | Kurilskoye | Sockeye | Shore | 24 | 22 | 2 | | 0 | |
|  |  | Ozernaya | Kurilskoye | Sockeye | Shore | 22 | 22 | 0 | | 0 | |
|  |  | South Bay | Kurilskoye | Sockeye | Shore | 24 | 24 | 0 | | 0 | |
|  |  | Etamink River | Kurilskoye | Sockeye | Stream | 24 | 10 | 7 | | 7 | |
|  |  | Gavrushka River | Kurilskoye | Sockeye | Stream | 24 | 2 | 9 | | 13 | |
|  |  | Kirushutk River | Kurilskoye | Sockeye | Stream | 24 | 4 | 5 | | 15 | |
|  |  | Vichenkiya River | Kurilskoye | Sockeye | Stream | 21 | 0 | 7 | | 14 | |
| 2 | Kronotsky Lake | Kronotsky Lake | Kronotskoye | Kokanee | Shore | 12 | 7 | 4 | | 1 | |
| 3 | Illiamna Lake | Fuel Dump Island | Illiamna | Sockeye | Shore | 14 | 2 | 5 | | 7 | |
|  |  | Knutson Bay | Illiamna | Sockeye | Shore | 21 | 11 | 8 | | 2 | |
|  |  | Woody Island | Illiamna | Sockeye | Shore | 20 | 2 | 7 | | 11 | |
|  |  | Chinkelyes Creek | Illiamna | Sockeye | Stream | 11 | 0 | 3 | | 8 | |
|  |  | Gibraltar Creek | Illiamna | Sockeye | Stream | 21 | 1 | 8 | | 12 | |
|  |  | Copper River | Illiamna | Sockeye | Stream | 20 | 0 | 5 | | 15 | |
| 4 | Mezadin Lake | Meziadin Beach | Nass | Sockeye | Shore | 48 | 43 | 4 | | 1 | |
|  |  | Tintina Creek | Nass | Sockeye | Stream | 19 | 6 | 7 | | 6 | |
|  |  | Hanna Creek | Nass | Sockeye | Stream | 24 | 8 | 10 | | 6 | |
| 5 | Gingit River | Gingit Creek | Nass | Sockeye | Stream | 23 | 2 | 4 | | 17 | |
| 6 | Babine Lake | Pierre Creek | Skeena | Kokanee | Stream | 15 | 0 | 0 | | 15 | |
| 7 | Tchesinkut Lake | Tchesinkut Lake | Skeena | Kokanee | Shore | 36 | 36 | 0 | | 0 | |
|  |  | Drew Creek | Skeena | Kokanee | Stream | 36 | 36 | 0 | | 0 | |
| 8 | Cowichan Lake | Cowichan Lake | Vancouver Island | Kokanee | Shore | 3 | 3 | 0 | | 0 | |
| 9 | Anderson-Seton Lakes | Anderson Lake | Fraser | Kokanee | Shore | 22 | 21 | 1 | | 0 | |
| 10 |  | Portage Creek | Fraser | Sockeye | Stream | 20 | 0 | 7 | | 13 | |
| 11 |  | Seton Lake | Fraser | Kokanee | Shore | 23 | 23 | 0 | | 0 | |
| 12 | Quesnel Lake | Quesnel Lake | Fraser | Kokanee | Shore | 27 | 27 | 0 | | 0 | |
| 13 | Nicola Lake | Upper Nicola River | Fraser | Kokanee | Stream | 24 | 0 | 9 | | 15 | |
| 14 | Adams Lake | Momich Creek | Fraser | Kokanee | Stream | 24 | 1 | 9 | | 14 | |
|  |  | Sinmax Creek | Fraser | Kokanee | Stream | 21 | 8 | 7 | | 6 | |
| 15 | Shuswap Lake | Eagle River | Fraser | Kokanee | Stream | 9 | 1 | 3 | | 5 | |
| 16 | Skaha Lake | Okanagan River | Columbia | Sockeye | Stream | 33 | 0 | 1 | | 32 | |
| 17 |  | Penticotin Channel | Columbia | Kokanee | Stream | 19 | 0 | 3 | | 16 | |
| 18 | Okanagan Lake | Okanagan Lake | Columbia | Kokanee | Shore | 144 | 141 | 3 | | 0 | |
|  |  | Mission Creek | Columbia | Kokanee | Stream | 136 | 0 | 28 | | 108 | |
| 19 | Wood Lake | Wood Lake | Columbia | Kokanee | Shore | 48 | 40 | 8 | | 0 | |
|  |  | Middle Vernon Creek | Columbia | Kokanee | Stream | 48 | 3 | 21 | | 24 | |
| 20 | Kalmalka Lake | Kalmalka Lake | Columbia | Kokanee | Shore | 32 | 29 | 3 | | 0 | |
|  |  | Coldstream Creek | Columbia | Kokanee | Stream | 32 | 3 | 9 | | 20 | |
| 21 | Christina Lake | Christina Lake | Columbia | Kokanee | Shore | 48 | 48 | 0 | | 0 | |
|  |  | Sanders Creek | Columbia | Kokanee | Stream | 48 | 0 | 7 | | 41 | |
| 22 | Kootenay Lake | Kootenay Lake (West Arm) | Columbia | Kokanee | Shore | 46 | 6 | 22 | | 18 | |
|  |  | Duhamel Creek (West Arm) | Columbia | Kokanee | Stream | 32 | 1 | 10 | | 21 | |
|  |  | Meadow Creek (North Arm) | Columbia | Kokanee | Stream | 22 | 0 | 5 | | 17 | |
| 23 | Redfish Lake* | Fishhook Creek | Columbia | Kokanee | Stream | 34 | 1 | 5 | | 28 | |
|  |  | Redfish Lake | Columbia | Sockeye | Shore | 99 | 99 | 0 | | 0 | |

*Allele frequency data from Nichols et al. (2016).

**Table S4.** Genes surrounding the LRRC9 gene in Atlantic Salmon, *Salmo salar* (assembly ICSASG_v2 - Chromosome ssa09).

| Gene | Description | Start | Stop |
| --- | --- | --- | --- |
| LOC106610988 | lateral signaling target protein 2 homolog | 24,469,376 | 24,529,117 |
| LOC106610987 | fibroblast growth factor receptor-like 1 | 24,532,072 | 24,597,035 |
| LOC106610986 | transforming acidic coiled-coil-containing protein 3-like | 24,603,180 | 24,615,295 |
| kif15 | kinesin family member 15 | 24,615,766 | 24,637,640 |
| LOC106610980 | putative ATP-dependent RNA helicase TDRD9 | 24,654,730 | 24,685,824 |
| LOC106610983 | protein RD3-like | 24,656,837 | 24,659,884 |
| rtn1 | reticulon 1 | 24,686,162 | 24,738,071 |
| LOC106610984 | uncharacterized | 24,697,938 | 24,703,205 |
| LOC106610979 | leucine-rich repeat-containing protein 9-like | 24,737,081 | 24,760,736 |
| LOC106610978 | uncharacterized | 24,794,590 | 24,801,845 |
| pcnxl4 | pecanex homolog 4 | 24,802,294 | 24,812,873 |
| dhrs7 | dehydrogenase/reductase 7 | 24,812,681 | 24,819,915 |
| LOC106610975 | protein phosphatase 1A-like | 24,820,320 | 24,837,750 |
| six6 | SIX homeobox 6 | 24,902,777 | 24,905,552 |
| LOC106610920 | piggyBac transposable element-derived protein 4-like | 24,914,086 | 24,916,725 |
| LOC106610972 | homeobox protein SIX4-like | 24,914,086 | 24,916,725 |
| six1 | SIX homeobox 1 | 24,949,211 | 24,951,236 |
| mnat1 | CDK activating kinase assembly factor | 24,965,289 | 25,024,032 |
